# Assessing the Effect of Simultaneous Combining of Transcranial Direct Current Stimulation and Transcutaneous Auricular Vagus Nerve Stimulation on the Improvement of Working Memory Performance in Healthy Individuals

**DOI:** 10.3389/fnins.2022.947236

**Published:** 2022-07-19

**Authors:** Rui Zhao, Zhao-Yang He, Chen Cheng, Qian-Qian Tian, Ya-Peng Cui, Meng-Ying Chang, Fu-Min Wang, Yao Kong, Hui Deng, Xue-Juan Yang, Jin-Bo Sun

**Affiliations:** ^1^School of Electronics and Information, Xi'an Polytechnic University, Xi'an, China; ^2^Intelligent Non-invasive Neuromodulation Technology and Transformation Joint Laboratory, Xidian University, Xi'an, China; ^3^Engineering Research Center of Molecular and Neuro Imaging of the Ministry of Education, School of Life Science and Technology, Xidian University, Xi'an, China; ^4^Guangzhou Institute of Technology, Xidian University, Xi'an, China

**Keywords:** tDCS, taVNS, working memory, spatial n-back, simultaneous joint stimulation

## Abstract

A previous study found that combining transcranial direct current stimulation (tDCS) and transcutaneous auricular vagus nerve stimulation (taVNS) could evoke significantly larger activation on a range of cortical and subcortical brain regions than the numerical summation of tDCS and taVNS effects. In this study, two within-subject experiments were employed to investigate its effects on working memory (WM). In experiment 1, the WM modulatory effects of tDCS over the left dorsolateral prefrontal cortex (DLPFC), taVNS, and simultaneous joint simulation of tDCS over the left DLPFC and taVNS (SJS-L) were compared among 60 healthy subjects. They received these three interventions between the baseline test and post-test in a random manner three times. In spatial 3-back task, there was a significant interaction between time and stimulations in the accuracy rate of matching trials (mACC, *p*=*0.018*). MACCs were significantly improved by SJS (*p* = *0.001*) and taVNS (*p* = *0.045*), but not by tDCS (*p* = *0.495*). Moreover, 41 subjects in the SJS group showed improvement, which was significantly larger than that in the taVNS group (29 subjects) and tDCS group (26 subjects). To further investigate the generalization effects of SJS, 72 students were recruited in experiment 2. They received tDCS over the right DLPFC, taVNS, simultaneous joint simulation of tDCS over the right DLPFC and taVNS (SJS-R), and sham stimulation in a random manner four times. No significant results were found, but there was a tendency similar to experiment 1 in the spatial 3-back task. In conclusion, combining tDCS and taVNS might be a potential non-invasive neuromodulation technique which is worthy of study in future.

## Introduction

Working memory (WM) is central to a number of higher order cognitive functions, the importance of which has been underlined by the field of cognitive psychology (Chiesa et al., 2011). On the one hand, the restricted amount of information that can be stored in WM is one of the central limitations of human cognition (Cowan, 2001), the differences of which among individuals are associated with variation in several important abilities, such as academic performance (Gathercole et al., 2003). On the other hand, WM impairment often occurs in a range of neuropsychiatric disorders, like schizophrenia (Winograd-Gurvich et al., 2006), and plays a crucial role in normal neurocognitive aging and the rapid cognitive deterioration associated with dementias, such as Alzheimer's disease (Grady, 2012). Previous researchers widely used drug therapies or behavior interventions to improve WM, whereas at present, non-invasive transcranial electrical stimulation (tES) techniques, such as transcranial direct current stimulation (tDCS), are gradually becoming the mainstream clinical treatment approaches (Constantinidis and Klingberg, 2016; Chase et al., 2020).

A number of studies have indicated that anodal tDCS placed on both left (Ke et al., 2019) and right (Giglia et al., 2014) dorsolateral prefrontal cortices (DLPFCs) would evoke immediate positive effects on WM performance in both healthy and clinical populations (Boggio et al., 2006; Giglia et al., 2014; Ke et al., 2019). Although the mechanism of tDCS is difficult to elucidate, researchers have suggested that tDCS might influence the central nervous system (CNS) through two mechanisms. First, tDCS may have a modulatory role on neural activity through the influence on network-level neural functions such as oscillatory dynamics (Liu et al., 2018; Chase et al., 2020). These effects would emerge from small changes in spike predictability and timing and may exert an effect of cognition *via* an influence on neural coding (McDonnell and Abbott, 2009). Second, at the cellular level, the changes of cognitive functions may arise from an impact of tDCS on membrane potentials and thus influence neural plasticity, that is, long-term potentiation (Kronberg et al., 2017). Through these two mechanisms, tDCS can exert effects on cognitive performance through a top-down mechanism. However, several recent meta-analysis studies casted doubt on the effects of tDCS on modulating WM (Hill et al., 2016; Sloan et al., 2021), which puts forward the further demand for more efficient stimulation protocols.

At the same time, transcutaneous auricular vagus nerve stimulation (taVNS) as an emerging cranial nerve stimulation method has gained ever-increasing scientific interests in a variety of cognition modulation and showed tremendous potential (Adair et al., 2020; Neuser et al., 2020). The vagus nerves, a kind of cranial nerve, belong to the peripheral nervous system (PNS) (Adair et al., 2020). The vagus nerves are intimately linked to perception and regulation of the CNS, with “bottom-up” functions in cognition and clinical disorders. It project to the nucleus tractus solitarii (NTS) in the medulla, before relayed further to other brainstem nuclei and higher order structures, including the thalamus, hippocampus, amygdala, and insula (Goehler et al., 2000; Saper, 2002). Recently, some brain imaging studies, such as functional magnetic resonance imaging (fMRI) studies, found that taVNS could influence a range of cortex and subcortex regions, including the contralateral postcentral gyrus, bilateral insula, frontal cortex, right operculum, left cerebellum, insula, hippocampus, amygdala, and thalamus (Yakunina et al., 2017; Badran et al., 2018). Moreover, behavioral studies have confirmed that taVNS could improve a series of cognitive functions, such as WM (Sun et al., 2021a), inhibitory control processes (Beste et al., 2016), and divergent thinking (Colzato et al., 2018). Considering similar modulatory effects and the complementary modulatory mechanism of tDCS and taVNS (top-down and bottom-up mechanisms) on cognition, the effects of their simultaneous joint stimulation with dual-path mechanisms are worthy of exploring.

Nowadays, the simultaneous joint stimulation methods have already been used to find an optimized strategy to promote brain activation, thereby improving cognitive functions. For instance, the synchronized intermittent theta burst stimulation (iTBS) and invasive vagus nerve stimulation (VNS) have provided a safe, feasible, and potentially effective way for depression treatment (George et al., 2020), while the anodal tDCS combined with repetitive peripheral nerve stimulation (rPNS) could promote motor hand recovery of the stroke patients (Sattler et al., 2015). Nevertheless, the simultaneous joint stimulation techniques do not always have positive effects. For example, researchers have explored the effect of tDCS combined with functional electrical stimulation (FES) on the activity of tibialis anterior muscles and the static balance of individuals with hemiparesis stemming from stroke, but there was no significant difference between the effects of tDCS alone and combined tDCS and FES (Fruhauf et al., 2017). Moreover, another study focused on the modulation of corticospinal excitability found that the peripheral nerve electrical stimulation (PES) combined with tDCS suppressed the effect of tDCS, which indicated that the PES might inhibit a slight membrane potential fluctuation of motor cortical neurons caused by tDCS (Adair et al., 2020). Considering the complexity of the aforementioned dual-path mechanism, the effects of combined tDCS and taVNS need to be further explored.

A recent brain imagining study has found that combining tDCS and taVNS induced significantly larger activation on a range of cortical and subcortical brain regions than the numerical summation of tDCS and taVNS effects, including the bilateral thalamus, pallidum, parahippocampal gyrus, dorsal raphe nucleus, substantia nigra, and periaqueductal gray matter (Sun et al., 2021b). Nevertheless, their synergistic effects on cognitive abilities, especially on WM, are still unknown. Thus, the current study employed two experiments with spatial and digit n-back tasks to compare the modulatory effects of tDCS, taVNS, and the combined stimulation of tDCS and taVNS (SJS) on WM performance. We hypothesized that this SJS intervention would be more effective in improving WM capacity than any single stimulation technique.

## Materials and Methods

### Experiment 1

In the first experiment, three stimulation conditions were set up, namely, tDCS over the left DLPFC (L-DLPFC tDCS), taVNS, and combined tDCS over the left DLPFC and taVNS (SJS-L). According to previous studies, there are some differences between the left and right DLPFCs (Barbey et al., 2013), and the anode on the left DLPFC would evoke significant modulatory effects (Dubreuil-Vall et al., 2019). Thus, we employed anodal left-DLPFC tDCS in the first experiment. In the standard taVNS protocol, electrodes were placed in the left cymba conchae with the anode outside (Neuser et al., 2020; Sun et al., 2021a), and the same protocol was employed in the present study.

#### Participants

A total of 60 healthy students at Xidian University were included in this experiment. All participants were right-handed, non-smokers, and had no history of neurological disease (e.g., epilepsy), psychiatric disorders (e.g., schizophrenia and depression), or brain damage. Since the present study focused on the modulatory effects on healthy participants, subjects who had any regular medication use were excluded. No participants reported ear injuries, drinking, or taking any medication 48 h before the experiment. Before the experiment, the participants were provided with information about the stimulation procedure and experimental protocols and signed the informed consent. The participants who successfully completed the experiment received corresponding remuneration, and they could withdraw from the experiment at any time if they did not wish to continue. Overall, three students did not complete all the three sessions because of personal reasons, and one student was excluded as the spatial 3-back matching accuracy rate before one stimulation condition was <30%. The final statistical analysis included 56 students (mean age 20.89 ± 1.92 years, range from 18 to 26 years; 31 female participants). The research was conducted in accordance with the Declaration of Helsinki and was approved by the institutional research ethics committee of the Xijing Hospital of the Air Force Medical University.

#### Experiment Design

**Figure 1:**
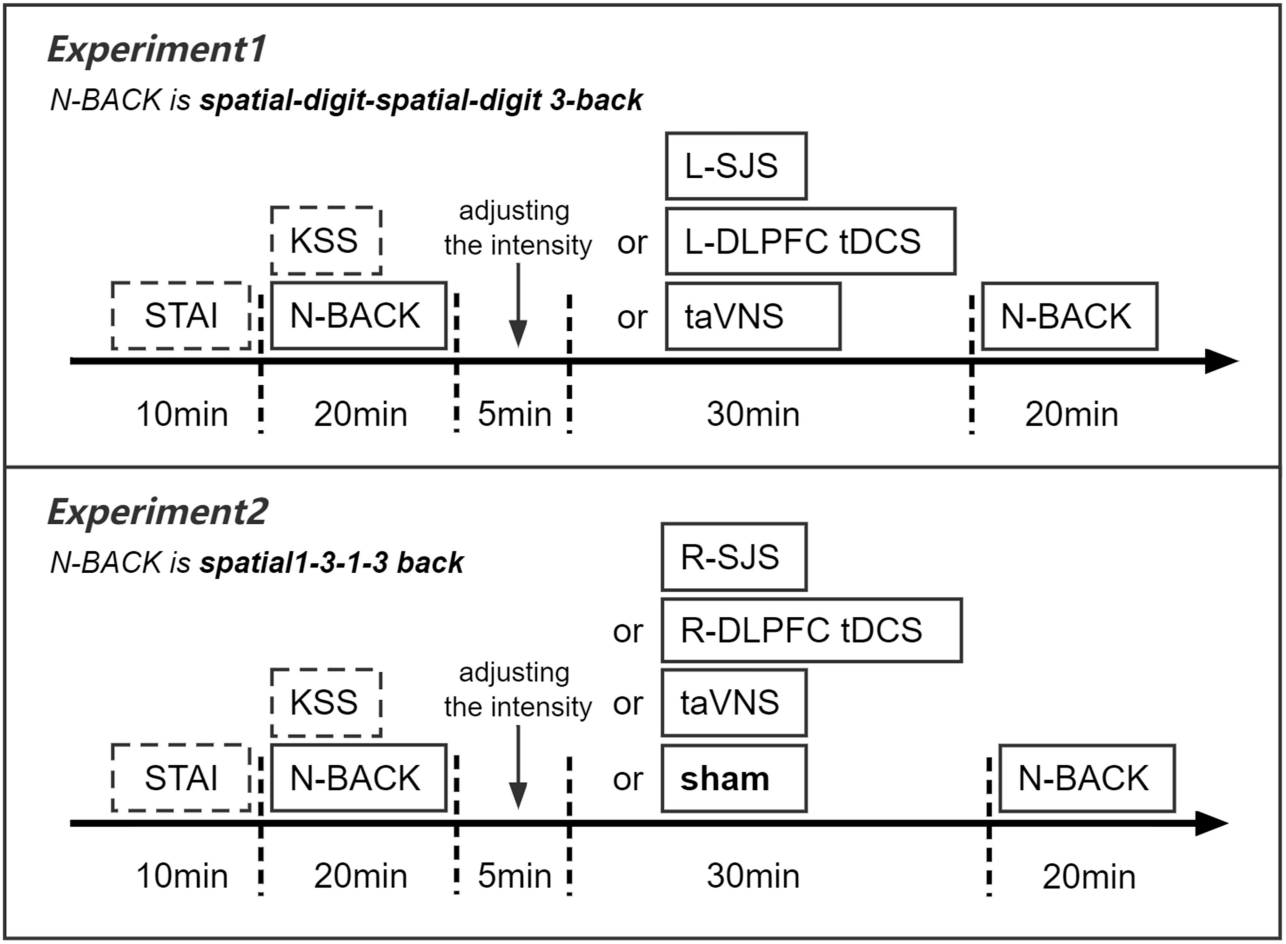
Design of experiments 1 and 2.

To test the facilitation of tDCS over the left DLPFC and its combined effects with taVNS, we employed a within-subjects design in this experiment, with each participant completing three separate sessions, which had different stimulation conditions, such as L-DLPFC tDCS, taVNS, and SJS-L. Every two sessions were separated by a period of 3 days, such that participants completed three different sessions at the first, fourth, and seventh days (Sun et al., 2021a). The stimulation orders were balanced between the participants. Before the formal experiment sessions, the participants first came to the laboratory to familiarize themselves with the experimental procedure and practice the behavioral tasks (see the section of “Behavioral task paradigm” for details). Then, each participant underwent three formal sessions with different stimulation conditions. Each session lasted about 90 min, with a 10-min pre-test of the State-Trait Anxiety Inventory (STAI; Cronbach's α 0.90) and Karolinska Sleepiness Scale (KSS), 10-min preparation of wearing stimulation equipment of tDCS and taVNS and a testing current intensity threshold of taVNS to reach a “moderate strong but not painful sensation,” a 20-min pre-test of behavioral tasks (baseline state), 30-min stimulation, and 20-min post-test of behavioral tasks (post-stimulation state) (Figure 1). In all three sessions, the stimulus electrodes of taVNS were placed at the left cymba conchae, and the standard wet sponge electrodes of tDCS were placed at the left frontal lobe and right orbitofrontal region (F3 and Fp2 in the 10–20 system) all the time, and it would always be in good contact during the entire experiment session.

#### tDCS and taVNS Equipment and Parameters

Electrical stimulation equipment used in this study was provided by XD-Kerfun Intelligent Non-invasive Neuromodulation Technology and Transformation Joint Laboratory, Xidian University, Xi'an, China, model BS-VNS-001, and was powered by a current source. The stimulator had two channels: one channel for taVNS and the other for tDCS. The tDCS channel was connected to two standard wet sponge electrodes (5 × 5 cm). The anode and cathode of tDCS were placed at the left frontal lobe and right orbitofrontal region (F3 and Fp2 in the 10–20 system), respectively. The current intensity of tDCS was set at 1 mA, with a ramp of 30 s (de Boer et al., 2021). The taVNS channel was connected to two silver chloride electrodes (outer diameter 7 mm). The anode and cathode of taVNS were both placed in the left cymba conchae, with the anode outside and 0.5 cm apart from the cathode. The electrical stimulation waveform was a single-phase rectangular pulse, with a pulse width of 500 μs and a frequency of 25 Hz (Neuser et al., 2020; Sun et al., 2021a; Shen et al., 2022). The current intensity was set according to the sensory threshold of each subject. The current was delivered with a cycle of 30 s on and 30 s off to avoid habituation (Neuser et al., 2020; Sun et al., 2021a).

Since perceived and tolerated stimulation intensities vary across participants, the current intensity was determined by each participant by using the threshold method to match the subjective experience of the stimulation. The processes were the same as used in our previous studies (Sun et al., 2021a). Briefly, the participants were asked to give direct feedback on their feeling of each stimulation intensity on a 10-point numerical rating scale (NRS), ranging from (1) no perception to (3) light tingling to (6) strong tingling to (10) intense pain. The stimulation started with an intensity of 0.1 mA and increased stepwise in 0.1 mA increments until the subject reported a slight feeling of pain (corresponding to 7 or higher on the subjective sensation scale), then decreased in 0.1 mA increments until 0.1 mA below the light tingling threshold (corresponding to 3 or lower on the subjective sensation scale). The protocol was repeated twice, and the average of the intensities rated as 5 (mild tingling) was used as the stimulation threshold.

#### Working Memory Tasks

In this experiment, there were two classical WM tasks (spatial 3-back WM task and digit 3-back WM task). Psychology experiment computer program E-Prime version 3.0 was employed to administer the tasks and record response accuracy and reaction time of all the participants.

The n-back task is one of the most frequently used paradigms in the assessment of WM capacity (Jarrold and Towse, 2006). The spatial and digit 3-back tasks in this experiment were organized according to a block design paradigm during which participants watched 72 stimuli in the task repeated in four blocks. During testing, the task 3-back order was quasi-randomized (spatial 3-back, digit 3-back, spatial 3-back, digit 3-back). Each 3-back level had the same number of “matching” (*n* = 24) and “non-matching” (*n* = 48) responses. Before each 3-back task was performed, the participants were required to complete a small practice of that 3-back, and they could not allow to start the formal experiment unless their accuracy rate reached more than 75%, and the average reaction time was <800 ms. In the formal task, each cycle consisted of a resting period of 5 min immediately followed by an activation block in which the 72 stimuli were presented one at a time (400 ms of exposure time), with an interstimulus interval of 1,600 ms (corresponding to 2,000 ms per trial). In the spatial 3-back task, the stimuli in the proposed task consisted of nine different nine-square matrices, with one of the squares marked with an ^“*.”^ Each trial was inserted as a picture format, with 257 × 257 pixels of width and height. Stimuli were presented in a pseudorandom order to avoid more than three consecutive trials of the same type. The participants were required to indicate whether each ^“*”^ was in the same location as three trials earlier. Response was forced choice and made with a keyboard, with “J” required for matches and “F” required for non-matches. The procedure of the digit 3-back task was the same as the spatial 3-back task, but the stimuli were changed from the site of ^“*”^ to nine Arabic numbers (1, 2, 3, 4, 5, 6, 7, 8, and 9). The font of each number was Times New Roman, and the font size was 72.

#### Data Analysis

Accuracy in matching trials (mACC), accuracy in mismatching trials (mismACC), and reaction time in accurate trials (aRT) on the 3-back tasks were used as the primary WM outcome measures. Permutation test-based one-way and two-way ANOVAs with 5,000 random samplings were employed to test the main effects of stimulation and the interaction between time and stimulation. First, one-way ANOVAs were used to confirm that stimulation conditions did not significantly differ in accuracy or response time at baseline (both *p* >0.05). Second, effects of stimulation on accuracy and reaction time were first assessed separately using 2 × 3 repeated measures ANOVAs with stimulation (SJS, L-DLPFC tDCS, and taVNS) and time (baseline and post-test) as the within-subject factors. Significant interaction effects were further explored *via* separate repeated measures ANOVAs for each stimulation conditions to examine changes over time (baseline and post-test). Additionally, one-way ANOVAs were used to compare the change from baseline (i.e., post-test) to pre-test scores (Δ-scores) between stimulation conditions. Analysis of Δ-scores allows for a direct comparison of whether changes in WM capacity significantly differed between stimulation conditions, and it was consistent with previous research examining transcranial electrical stimulation-reduced changes in WM performance (Murphy et al., 2020). Pairwise comparisons with Bonferroni correction were used to explore any significant main effects. Third, for WM outcome measures which displayed significant changes over time, we examined the consistency of improvements induced by SJS, L-DLPFC tDCS, and taVNS by comparing the proportion of participants in each stimulation group who demonstrated improvements compared with baseline performance. Then, a chi-square test was used to compare whether the proportion of participants displaying improvements was significantly different among the three stimulation groups. Finally, for WM outcome measures which showed larger improvements in the SJS group than L-DLPFC tDCS and taVNS groups, we further compared its effects with the numerical summation effects of L-DLPFC tDCS and taVNS (i.e., L-DLPFC tDCS + taVNS).

### Experiment 2

Since functions of left and right DLPFCs are different, to a certain extent, the second experiment was developed to test the specificity of tDCS sites. Thus, in this experiment, there are four stimulation conditions, including tDCS over the right DLPFC (R-DLPFC tDCS), taVNS, combined R-DLPFC tDCS and taVNS (SJS-R), and sham stimulations. Additionally, some studies suggested that the neural state created by tasks with different difficulties might be differentially susceptible to modulation by tDCS and thus might yield different outcomes in behavior (Onton et al., 2005; Chase et al., 2020). Therefore, based on experiment 1, we adopt spatial 1-back and 3-back tasks to test the specificity of cognitive loads.

#### Participants

A total of 72 healthy students at Xidian University were included, and the recruitment criterion was the same as in experiment 1. Overall, five students did not complete all the four sessions of the experiment because of their time constraints, three students were excluded because of low accuracy rates in the spatial 3-back task (at least the pre-test of one session was <30%), and one student was excluded due to missing stimulation order data. Finally, 63 subjects were included in the statistical analysis (mean age 20.76 ± 1.58 years, range from 18 to 25 years; 33 female participants).

#### Experiment Design

To further test the specificity of stimulation sites and cognitive loads, this within-subject experiment included four stimulation conditions, namely, SJS-R (simultaneous combined tDCS over the right DLPFC with taVNS), R-DLPFC tDCS, taVNS, and sham, with spatial 1-back and 3-back tasks. The basic procedure was the same as the first experiment, except that each participant needed to implement four independent sessions (Figure 1).

#### tDCS and taVNS Equipment and Parameters

Apart from placement of the anode and cathode of tDCS at the right frontal lobe and left orbitofrontal regions (F4 and Fp1 in the 10–20 system), respectively, all the details of stimulation were the same as experiment 1.

#### Working Memory Tasks

In this experiment, we employed 1-back and 3-back tasks with spatial stimuli. There were four blocks (1-back, 3-back, 1-back, and 3-back) with 72 experiment trials in each block. Each block was separated by a 30-sec rest period. Before these four blocks, there was a training block with 16 trials for 1-back and 3-back task, respectively. The participants were instructed to press “F” when the site of the symbol (^“*”^) was the same as the 1-back or 3-back trials earlier (i.e., “matching” trial), otherwise press “J.” Each trial was inserted as a picture format, with 257 × 257 pixels of width and height. Totally, one-third of the trials were matching. Training trials were conducted before experiment trials, and the participants could not start the formal experiment unless their training accuracy rate reached more than 75% and the average reaction time was <1,000 ms. Psychology experiment computer program E-Prime version 3.0 was employed to administer the tasks and record response accuracy and reaction time of all the participants.

#### Data Analysis

The data analysis processes were similar to experiment 1, including permutation test-based one-way and two-way repeated measures ANOVAs to test the difference in baseline performance among the four stimulation conditions and the main effects of time and stimulation and their interaction effects, and a chi-square test to compare the proportion of participants in each stimulation group who demonstrated improvements in accuracy which were greater than simple practice effects, defined as the mean change in performance displayed by the sham group from baseline to post-test. For the outcome measures, which exhibited significant interaction between time and stimulation, we compared the effects of right SJS with the numerical summation effects of tDCS and taVNS (tDCS + taVNS). Finally, to replicate the positive results in experiment 1 and investigate the specificity of stimulation sites, we employed the full analysis in mACC of the spatial 3-back task.

## Results

### Experiment 1

#### Subjective Sensation

At the end of each session, the participants used the NRS to quantify their pain sensation during the previous stimulation condition. There was no significant difference in the subjective sensation (using the NRS) evoked by the three stimulation conditions.

#### Spatial 3-Back Working Memory Performance

##### Reaction Time in Accuracy Trials (aRT)

There were no significant difference in the pre-test among three different stimulation groups [*F*_(2,110)_ = 0.56, *p* = 0.565]. A significant main effect of time was observed [*F*_(1,55)_ = 34.23, *p* < 0.001], suggesting that participants' performance was much faster in the post-test than in baseline. No significant main effect of stimulation conditions and interaction between stimulation and time was found (Table 1). Considering the consistency of improvements, 44 subjects (78.6%) in the SJS-L group, 37 subjects (66.1%) in the L-DLPFC tDCS group, and 48 subjects (85.7%) in the taVNS group showed improvements, and the difference in the improvement proportion between three groups was significant [${\text{χ}}_{\left(2\right)}^{2}$ = 6.21, *p* = 0.045]. However, no significant difference was found between the SJS-L group and the L-DLPFC tDCS group [${\text{χ}}_{\left(1\right)}^{2}$ = 1.61, *p* = 0.174] or taVNS group [${\text{χ}}_{\left(1\right)}^{2}$ = 0.55, *p* = 0.458], while the taVNS group evoked a larger improvement proportion than the L-DLPFC tDCS group [${\text{χ}}_{\left(1\right)}^{2}$ = 4.89, *p* = 0.027].

**Table 1:** Means, standard deviations, and two-way ANOVA results of WM tasks in experiment 1.

**Descriptive statistics**		**Spatial 3-back**			**Digit 3-back**		
		**aRT**	**mACC**	**mismACC**	**aRT**	**mACC**	**mismACC**
**Time**	**Stim**	**Mean ±SD**	**Mean ±SD**	**Mean ±SD**	**Mean ±SD**	**Mean ±SD**	**Mean ±SD**
Pre-test	SJS	662.8 ± 150.4	0.816 ± 0.154	0.945 ± 0.074	572.2 ± 110	0.865 ± 0.137	0.959 ± 0.081
	tDCS	650.5 ± 154.1	0.822 ± 0.143	0.95 ± 0.056	561.4 ± 116.2	0.875 ± 0.107	0.971 ± 0.032
	taVNS	649 ± 154.3	0.825 ± 0.954	0.156 ± 0.049	557.4 ± 122.8	0.859 ± 0.158	0.967 ± 0.05
Post-test	SJS	626.2 ± 142.7	0.877 ± 0.103	0.961 ± 0.064	550.1 ± 110.3	0.884 ± 0.109	0.968 ± 0.052
	tDCS	617 ± 143.1	0.836 ± 0.126	0.961 ± 0.048	537.7 ± 110.3	0.874 ± 0.11	0.973 ± 0.032
	taVNS	598.8 ± 137.2	0.856 ± 0.126	0.961 ± 0.052	530.9 ± 114.5	0.881 ± 0.132	0.971 ± 0.033
Permutaion-based RM ANOVA test							
Time		*F*_(1,55)_ = 34.23^***^	*F*_(1,55)_ = 20.51^***^	*F*_(1,55)_ = 18.86^***^	*F*_(1,55)_ = 32.02^***^	*F*_(1,55)_ = 3.94	*F*_(1,55)_ = 3.12
Stimulation		*F*_(2,110)_ = 1.14	*F*_(2,110)_ = 1.39	*F*_(2,110)_ = 0.307	*F*_(2,110)_ = 0.95	*F*_(2,110)_ = 0.09	*F*_(2,110)_ = 0.99
Time × Stimulation		*F*_(2,110)_ = 1.13	*F*_(2,110)_ = 6.27^*^	*F*_(2,110)_ = 1.03	*F*_(2,110)_ = 0.10	*F*_(2,110)_ = 1.63	*F*_(2,110)_ = 0.40

* *Corrected p-value smaller than 0.05*.

*** *Corrected p-value smaller than 0.001*.

*All p-values were corrected by Bonferroni correction. SJS is the simultaneous joint simulation of tDCS over the left DLPFC and taVNS; SD means standard deviation; RM ANOVA means repeated measures analysis of variance. The bold results are significant results*.

##### Accuracy Rate in Matching Trials (mACC)

**Figure 2:**
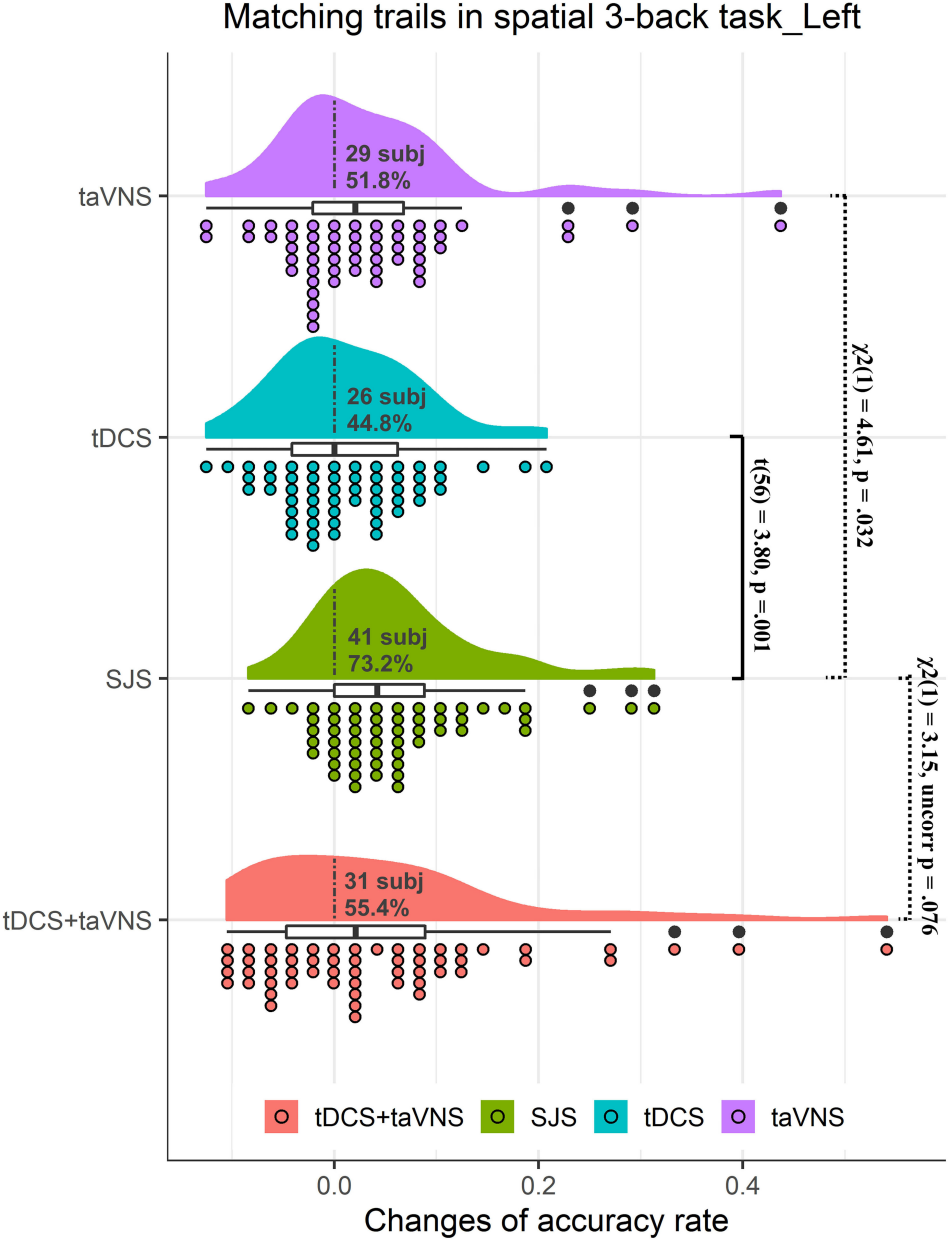
Rain cloud plots of accuracy rate in matching trials (mACC) of the spatial 3-back task in experiment 1. Clouds and box plots represent the distribution of Δ-scores between the post-test and pre-test in taVNS, tDCS, SJS-L (simultaneous joint simulation of tDCS and taVNS), and tDCS+taVNS (the numerical summation effects of tDCS and taVNS) groups. Plots represent the Δ-score of each participant in the four conditions. Dotted lines separate positive change data with negative change data, and the number and proportion of subjects with improvements are presented in the clouds. The significant difference between tDCS and SJS-L groups and the improvement proportion difference between SJS-L and taVNS and tDCS+taVNS are presented in the figure.

There was no significant difference between the three groups in baseline performance [*F*_(2,110)_ = 0.24, *p* = 0.797]. The main effect of time was significant [*F*_(1,55)_ = 20.51, *p* < 0.001], suggesting that participants had a higher accuracy rate in the post-test than in baseline. The interaction between time and stimulation conditions was significant [*F*_(2,110)_ = 6.27, *p* = 0.018] (Table 1). *Post-hoc* analysis revealed that mACC significantly increased following SJS [*t*_(55)_ = 5.64, *p* = 0.001] and taVNS [*t*_(55)_ = 2.46, *p* = 0.045], but not L-DLPFC tDCS [*t*_(55)_ = 1.46, *p* = 0.442] (Table 1). Direct comparison of stimulation conditions using accuracy Δ-scores revealed significant group differences between SJS-L and L-DLPFC tDCS [*t*_(55)_ = 3.80, *p* = 0.001], but no significant difference between SJS-L and taVNS was observed [*t*_(55)_ = 2.09, *p* = 0.114] (Table 2). Considering the consistency of improvements, 41 students (70.7%), 26 students (44.8%), and 29 students (50.0%) in the SJS-L group, L-DLPFC tDCS group, and taVNS group showed improvements, respectively, and there was a significant difference in the improvement proportion between the three groups [${\text{χ}}_{\left(2\right)}^{2}$ = 9.19, *p* = 0.010], and the difference between the SJS-L group and L-DLPFC tDCS group [${\text{χ}}_{\left(1\right)}^{2}$ = 7.28, *p* = 0.007] and taVNS group [${\text{χ}}_{\left(1\right)}^{2}$ = 4.61, *p* = 0.032] was significant. However, the synergistic effects of SJS-L was not significantly larger than L-DLPFC tDCS+taVNS [*t*_(55)_ = 0.94, *p* = 0.351], while there was a significant tendency in the improvement proportion between these two groups [41 students vs. 31 students, ${\text{χ}}_{\left(1\right)}^{2}$ = 3.15, *p* = 0.072] (Figure 2).

**Table 2:** *Post-hoc* of significant time–stimulation interactions.

**Task**	**Comparison**	**Group**	**mACC**			
			**Estimate**	**SD**	***t*-test**	*p*-value^*^
Spatial 3-back	Post-test *vs*. pre-test	SJS	0.061	0.081	*t*_(55)_ = 5.64	**0.001**
		tDCS	0.013	0.069	*t*_(55)_ = 1.46	0.429
		taVNS	0.032	0.096	*t*_(55)_ = 2.46	**0.045**
	Group vs. Group	SJS *vs*. tDCS	0.048	0.094	*t*_(55)_ = 3.80	**0.001**
		SJS *vs*. taVNS	0.029	0.105	*t*_(55)_ = 2.09	0.120
		tDCS *vs*. taVNS	0.018	0.105	*t*_(55)_ = −1.29	0.633

* *All p-values were corrected by Bonferroni correction. mACC is the accuracy rate in matching trials; SJS is the simultaneous joint simulation of tDCS over the left DLPFC and taVNS; SD means standard deviation. The bold results are significant results*.

##### Accuracy Rate in Mismatching Trials (mismACC)

There was no significant difference between the three groups in baseline performance [*F*_(2,110)_ = 0.73, *p* = 0.495]. The main effect of time was significant [*F*_(1,55)_ = 18.86, *p* = 0.001], and the accuracy of the post-test was higher than that of baseline. The interaction between time and stimulation was not significant (Table 1). Considering the consistency in improvements, 31 students (53.4%), 29 students (50.0%), and 28 students (48.3%) in the SJS-L group, L-DLPFC tDCS group, and taVNS group showed improvements, respectively, and there was no significant difference in the improvement proportion between the three groups [${\text{χ}}_{\left(2\right)}^{2}$ = 0.33, *p* = 0.846].

#### Digit 3-Back Working Memory Performance

##### Reaction Time in Accuracy Trials (aRT)

There was no significant difference between the three groups in baseline performance [*F*_(2,110)_ = 0.60, *p* = 0.552]. Except for the main effect of time [*F*_(1,55)_ = 32.02, *p* < 0.001], no significant effects were found in this outcome measure (Table 1). Considering the consistency of improvements, 38 students (67.9%), 42 students (75.0%), and 38 students (67.9%) in the SJS-L group, L-DLPFC tDCS group, and taVNS group showed improvements, respectively, and there was no significant difference in the improvement proportion between the three groups [${\text{χ}}_{\left(2\right)}^{2}$ = 0.91, *p* = 0.634].

##### Accuracy Rate in Matching Trials (mACC)

No significant difference among three groups in baseline performance was found [*F*_(2,110)_ = 0.59, *p* = 0.556]. And no significant results were observed in this outcome measure (Table 1).

##### Accuracy Rate in Mismatching Trials (mismACC)

No significant difference among three groups in baseline performance was found [*F*_(2,110)_ = 0.89, *p* = 0.414]. No significant results were observed in this outcome measure (Table 1).

### Experiment 2

#### Subjective Sensation

At the end of each session, the participants used the NRS to quantify their pain sensation during the previous stimulation condition. There was no significant difference in the subjective sensation (using the NRS) evoked by the three stimulation conditions (SJS-R, R-DLPFC tDCS, and taVNS).

#### Spatial 1-Back Working Memory Performance

##### Reaction Time in Accuracy Trials (aRT)

No significant difference in baseline performance among the four stimulation conditions was observed [*F*_(3,186)_ = 1.70, *p* = 0.168]. The main effect of time was significant [*F*_(1,62)_ = 46.93, *p* = 0.001]. The interaction between time and stimulation was not significant (Table 3). Taking the average change of the sham group as the threshold, 36 students (57.1%), 31 students (49.2%), and 25 students (40.0%) in SJS-R, R-DLPFC tDCS, and sham groups showed improvements, respectively, and there was no significant difference in the improvement proportion between the three groups [${\text{χ}}_{\left(3\right)}^{2}$ = 3.94, *p* = 0.268].

**Table 3:** Means, standard deviations, and two-way ANOVA results of WM tasks in experiment 2.

**Descriptive statistics**		**Spatial 1-back**			**Spatial 3-back**		
		**aRT**	**mACC**	**mismACC**	**aRT**	**mACC**	**mismACC**
**Time**	**Stim**	**Mean ±SD**	**Mean ±SD**	**Mean ±SD**	**Mean ±SD**	**Mean ±SD**	**Mean ±SD**
Pre-test	SJS	480.4 ± 92.0	0.911 ± 0.064	0.974 ± 0.023	604 ± 153.9	0.778 ± 0.142	0.941 ± 0.067
	tDCS	474.1 ± 79.9	0.892 ± 0.095	0.981 ± 0.019	594.2 ± 170.2	0.787 ± 0.147	0.971 ± 0.945
	taVNS	485.8 ± 87.4	0.904 ± 0.083	0.978 ± 0.023	606.1 ± 161.3	0.781 ± 0.151	0.938 ± 0.07
	Sham	467.8 ± 86.5	0.892 ± 0.094	0.977 ± 0.023	583.3 ± 156	0.805 ± 0.119	0.955 ± 0.063
Post-test	SJS	463.6 ± 86.5	0.889 ± 0.083	0.983 ± 0.016	579 ± 147.4	0.818 ± 0.133	0.949 ± 0.058
	tDCS	455.3 ± 66.6	0.892 ± 0.083	0.983 ± 0.016	572.7 ± 149.7	0.798 ± 0.132	0.952 ± 0.052
	taVNS	469.6 ± 88.6	0.072 ± 0.903	0.965 ± 0.087	588.9 ± 153.4	0.814 ± 0.951	0.13 ± 0.07
	Sham	452.7 ± 82.4	0.883 ± 0.112	0.981 ± 0.025	562.5 ± 149.4	0.813 ± 0.123	0.952 ± 0.06
Permutaion-based RM ANOVA test							
Time		*F*_(1,62)_ = 46.93^***^	*F*_(1,62)_ = 2.31	*F*_(1,62)_ = 0.03	*F*_(1,62)_ = 18.60^***^	*F*_(1,62)_ = 19.63^***^	*F*_(1,62)_ = 7.49^*^
Stimulation		*F*_(3,186)_ = 2.39	*F*_(3,186)_ = 1.55	*F*_(3,186)_ = 1.94	*F*_(3,186)_ = 1.31	*F*_(3,186)_ = 0.69	*F*_(3,186)_ = 1.04
Time × Stimulation		*F*_(3,186)_ = 0.10	*F*_(3,186)_ = 0.86	*F*_(3,186)_ = 2.07	*F*_(3,186)_ = 0.13	*F*_(3,186)_ = 2.51	*F*_(3,186)_ = 1.88

* *Corrected p-value smaller than 0.05*.

*** *Corrected p-value smaller than 0.001*.

*All p-values were corrected by Bonferroni correction. SJS is the simultaneous joint simulation of tDCS over the left DLPFC and taVNS; SD means standard deviation; RM ANOVA means repeated measures analysis of variance. The bold results are significant results*.

##### Accuracy Rate in Matching Trials (mACC)

No significant difference in baseline performance among the four stimulation conditions was observed [*F*_(3,186)_ = 1.24, *p* = 0.296]. Also no significant results were observed in this outcome measure (Table 3).

##### Accuracy Rate in Mismatching Trials (mismACC)

No significant difference in baseline performance among the four stimulation conditions was observed [*F*_(3,186)_ = 1.34, *p* = 0.263]. Also no significant results were observed in this outcome measure (Table 3).

#### Spatial 3-Back Working Memory Performance

##### Reaction Time in Accuracy Trials (aRT)

No significant difference in baseline performance among the four stimulation conditions was observed [*F*_(3,186)_ = 0.83, *p* = 0.479]. The main effect of time was significant [*F*_(1,62)_ = 18.60, *p* = 0.001]. The interaction between time and stimulation was not significant (Table 3). Taking the average change in the sham group as the threshold, 34 students (54.0%), 33 students (52.4%), and 34 students (54.0%) in SJS-R, R-DLPFC tDCS, and sham groups showed improvements, respectively, and there was no significant difference in the improvement proportion among three groups [${\text{χ}}_{\left(3\right)}^{2}$ = 0.13, *p* = 0.988].

##### Accuracy Rate in Matching Trials (mACC)

**Figure 3:**
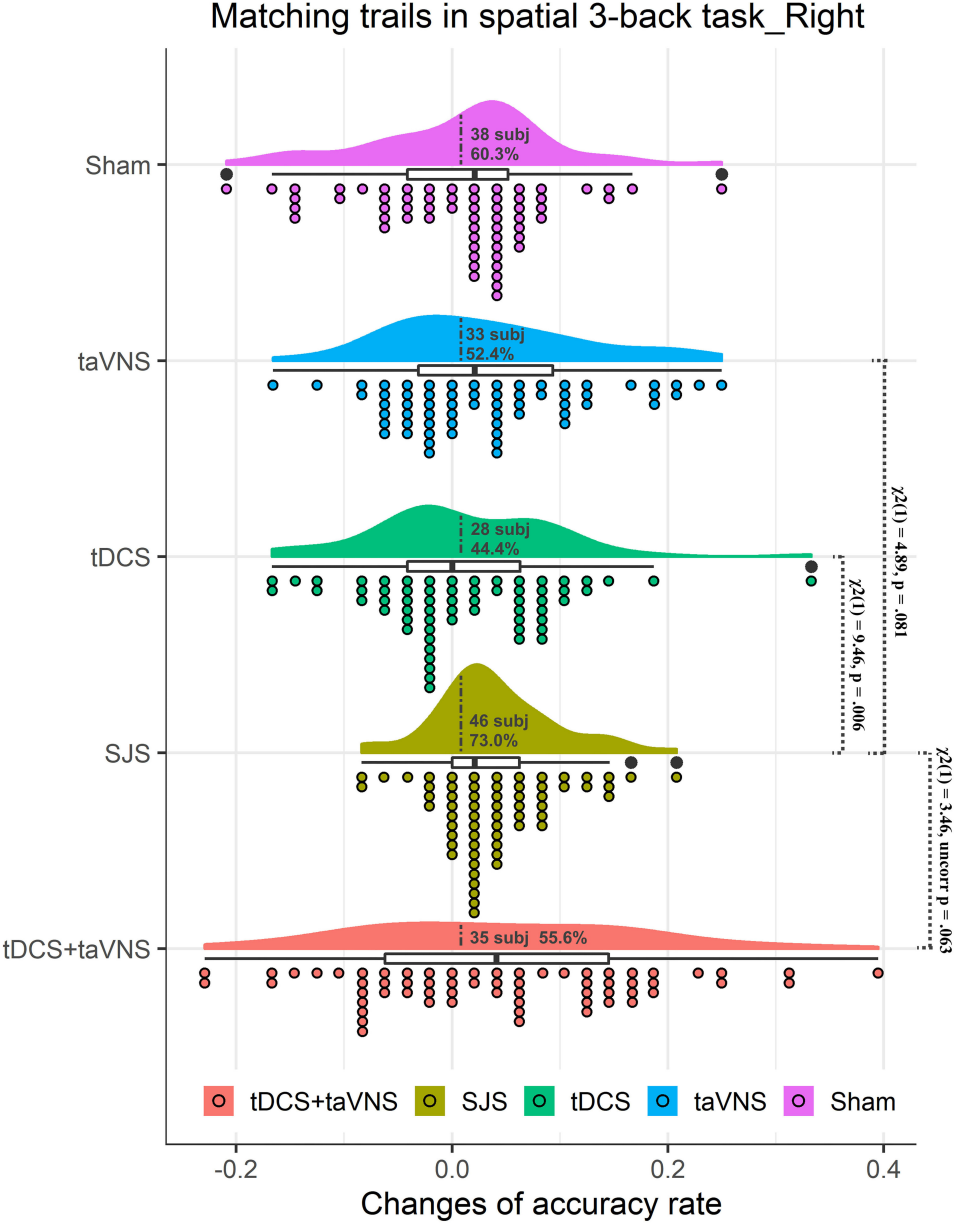
Rain cloud plots of accuracy rate in matching trials (mACC) of the spatial 3-back task in experiment 2. Clouds and box plots represent the distribution of Δ-scores between the post-test and pre-test in taVNS, tDCS, SJS-R (simultaneous joint simulation of tDCS and taVNS), and tDCS+taVNS (the numerical summation effects of tDCS and taVNS) groups. Plots represent the Δ-score of each participant in the four conditions. Dotted lines separate positive change data with negative change data, and the number and proportion of subjects with improvements are presented in the clouds. The significant difference between tDCS and SJS-R groups and the improvement proportion difference between SJS-R and taVNS and tDCS+taVNS are presented in the figure.

No significant difference in baseline performance among four stimulation conditions was observed [*F*_(3,186)_ = 1.29, *p* = 0.279]. The main effect of time was significant [*F*_(1,62)_ = 19.63, *p* = 0.001]. The main effect of stimulation and interaction effect between stimulation and time was not significant. However, there was a tendency similar to experiment 1. To be specific, mACCs were significantly improved by SJS-R (*p* = 0.001) and taVNS interventions (*p* = 0.026), but not by R-DLPFC tDCS (*p* = 1.00) or sham (*p* = 1.00) (Table 3 and Figure 3). Taking the average change of the sham group as the threshold (0.008), 46 students (73.0%), 28 students (44.4%), and 33 students (52.4%) in SJS-R, R-DLPFC tDCS, and taVNS groups showed improvements, respectively, and there was a significant difference in the improvement proportion between the four conditions [${\text{χ}}_{\left(3\right)}^{2}$ = 11.48, *p* = 0.009]. The proportion of participants in the SJS-R group who demonstrated improvements was significantly larger than that in the tDCS group [${\text{χ}}_{\left(1\right)}^{2}$ = 9.46, *p* = 0.006], whereas there was only a significant tendency between the SJS-R group and taVNS group [${\text{χ}}_{\left(1\right)}^{2}$ = 4.89, *p* = 0.081], or R-DLPFC tDCS group and taVNS group [${\text{χ}}_{\left(1\right)}^{2}$ = 0.51, *p* = 1.00]. The synergistic effects of SJS was not significantly larger than those of R-DLPFC tDCS+taVNS [*t*_(62)_ = 0.22, *p* = 0.826], while there was a significant tendency in the improvement proportion between these two groups [46 students vs. 35 students, ${\text{χ}}_{\left(1\right)}^{2}$ = 3.46, *p* = 0.063] (Figure 3).

##### Accuracy Rate in Mismatching Trials (mismACC)

No significant difference in baseline performance among the four stimulation conditions was observed [*F*_(3,186)_ = 2.05, *p* = 0.109]. The main effect of time was significant [*F*_(1,62)_ = 7.49, *p* = 0.041]. The interaction between time and stimulation was not significant (Table 3). Taking the average change of the sham group as the threshold, 39 students (61.9%), 42 students (66.7%), and 42 students (66.7%) in SJS-R, R-DLPFC tDCS, and sham groups showed improvements, respectively; however, there was a significant tendency in the improvement proportion between the three groups [${\text{χ}}_{\left(3\right)}^{2}$ = 7.56, *p* = 0.056].

## Discussion

The aim of the current study was to directly compare the cognitive modulated effects of tDCS, taVNS, and their combined stimulations (SJS) as neuromodulation tools for enhancing WM in healthy adults. First, we replicated the positive effects of taVNS on spatial 3-back task accuracy in our previous studies (Sun et al., 2021a), while no significant results were found in the tDCS group. However, when delivered using the common stimulation parameters (Sun et al., 2021a,b), we found that combining tDCS over the left or right DLPFC and taVNS, and single taVNS could significantly improve spatial 3-back WM task accuracy, whereas no significant cognitive effects were observed following anodal tDCS over the left or right DLPFC, or sham stimulation. Moreover, SJS induced more consistent improvements in WM accuracy than tDCS, taVNS, or their numerical summation effects.

To the best of our knowledge, this is the first study to explore the synergistic effects of tDCS and taVNS on WM performance and provides the evidence showing the SJS technique to be more effective than anodal tDCS for enhancing WM performance in healthy adults. The results of the current study are partly consistent with those of our previous research (Sun et al., 2021b), which found that SJS could evoke extensive activation in multiple brain networks, and in several regions, the activation effects were even stronger than those in the numerical summation of the activation by tDCS and taVNS. Recently, peripheral nerve arousal and vigilance were proposed as another possible and important mechanism of tDCS (van Boekholdt et al., 2021). Indeed, as tDCS is applied directly on the skin, and the currents need to pass through the scalp before reaching the cerebral cortex, localized peripheral nerves that distribute in the scalp, such as occipital nerve and the trigeminal nerve endings are exposed to much higher electric field strengths than the underlying cortices (Adair et al., 2020; van Boekholdt et al., 2021). Therefore, tDCS might exert effects through both transcranial (top-down) and transcutaneous (bottom-up) mechanism, which can be regarded as a simultaneous joint with dual-path mechanism to some extent. Thus, the combination with taVNS, one kind of peripheral nerve stimulation technique, might reinforce this simultaneous joint mechanism of tDCS and evoked better cognitive modulatory effects.

It is worth noting that in addition to improving the WM task accuracy rate, SJS induced more consistent improvements in WM accuracy than tDCS, taVNS, or their numerical summation effects. In the current study, only 44.8% participants in the L-DLPFC tDCS group and 44.4% students in the R-DLPFC tDCS group demonstrated improvements in spatial 3-back task accuracy. Consistent with this, Murphy et al. (2020) found that only 31.25% of participants in the tDCS group displayed improvements in task accuracy in their study (Murphy et al., 2020). Moreover, effects of tDCS appear to be highly variable at the individual level in previous studies, with experiment research one meta-analysis suggesting that only 16% of subjects showed the desired outcome in cognitive studies (Jacobson et al., 2012). Additionally, 50.0 and 52.4% participants in taVNS group displayed improvements, which suggested that taVNS facilitated WM performance with a relatively high individual difference. Supporting our hypothesis, combining tDCS with taVNS could largely increase improvement consistency. The underlying mechanisms of these improvements are currently unknown. A possible explanation of our results is that the modulatory effects of tDCS on neural activity depend on the “baseline” neural activities, which are different in individuals, while taVNS can influence neural baseline activities, making them sensitive to tDCS intervention. As it is known, afferent fibers from vagus nerves travel to the brainstem, where they project to the NTS (Adair et al., 2020). The information of NTS is integrated in the reticular formation (i.e., a large network of nerves with nuclei clusters throughout the brainstem) and further participates in the ascending reticular activating system (ARAS): a system that contains a set of nuclei that release neurotransmitters in the cortex, both directly and through thalamic relays (Schwartz and Kilduff, 2015). The ARAS can exert its influence on many basic behavioral processes through these nuclei, such as locus coeruleus (LC) and the main source of norepinephrine (NE) in the brain (Couto et al., 2006; Tyler et al., 2015). NE is known to increase cortical excitability, drive synaptic plasticity (Kuo et al., 2017b), and modulate cognition (Sara, 2009). Importantly, the effects of NE have already been reported as a mediator of tDCS effects (Monai et al., 2016; Kuo et al., 2017a). Thus, taVNS might induce the release of NE and modulate the cortical excitability, which would make neural activity more sensitive to tDCS intervention.

Beyond our expectation, we did not observe significant improvements in WM performance in both L-DLPFC tDCS and R-DLPFC tDCS groups, with highly individual differences. However, existing evidence for the facilitatory effects of anodal tDCS over the DFPFC on WM performance in both healthy and clinical cohorts is inconsistent, with several recent experiment research (Murphy et al., 2020) and meta-analyses (Brunoni and Vanderhasselt, 2014; Hill et al., 2016; Mancuso et al., 2016; Sloan et al., 2021) showing that the improvement of tDCS on WM capacity is modest and variable. It might cause by the effect of tDCS on neural plasticity, which could facilitate task training efficiency *via* improve task encoding but would not influence task performance directly (Simonsmeier et al., 2018). Thus, the WM enhancement potential of tDCS probably lies in its use during training (Mancuso et al., 2016).

From experiment 2, we further found that the synergistic effects of tDCS and taVNS on WM performance depended on task difficulty but not on the stimulation site of tDCS. Evidence is emerging that links tDCS-related improvement in task performance to neural oscillations. However, the neural state created by different tasks might be differentially susceptible to tDCS modulation and thus might yield different outcomes in behavior (Bikson et al., 2013). A difficult working memory task might elicit increases in the theta frequency band (~4–7 Hz), which is more sensitive to tDCS modulatory effects (Onton et al., 2005). For example, Reinhart et al. have found that tDCS enhanced behavioral performance on adaptive control tasks, specifically on tasks with increased cognitive demands, which might be associated neural oscillatory in the theta power (Reinhart et al., 2015). Consistent with these findings, the current study found that the modulatory effects of SJS were found in the spatial 3-back task accuracy but not in spatial 1-back tasks.

However, there are still some limitations of the current study and require further researchers in future. First, although results from the present study revealed a stronger modulatory effect of the combined tDCS and taVNS technique, especially in improvement consistency, it was not clear how they interacted in the cortex and influence individuals' WM performance, which needs more research with physiological signals in future. Second, considering the synergistic effects of SJS on brain activation that not only significantly larger than single tDCS and taVNS but also larger than their numerical summation effects (Sun et al., 2021b), further studies need to focus on which behavioral performance is sensitive to these brain changes and can be modulated by the synergistic effects of SJS. Third, since healthy subjects are easier to recruit than patients and it is important to testify technique's effects before applying in clinical experiments, the participants in the current study are healthy students. However, it is important to test the effects of SJS in clinical cohorts or old populations in future. Finally, research in future needs to focus on the influence of stimulation parameters, such as the different effects of anode and cathode, which might accelerate the application of tDCS, taVNS, and their combined stimulation.

## Conclusion

In summary, the current study showed a larger facilitation on WM memory of combined tDCS and taVNS than single tDCS and taVNS techniques, with a better tendency in increasing the consistency of improvements than the numerical summation of tDCS and taVNS, which makes it of important clinical significance. Further investigating its effects, safety, and specification may promote its clinical application. Additionally, combined stimulation techniques put a novel thought in non-invasive neuromodulation as tDCS combined with peripheral nerve stimulation showed greater effects on the motor recovery after stroke (Sattler et al., 2015); tDCS combined with transcranial random noise stimulation (tRNS), another transcranial stimulation, exhibited better facilitation on WM (Murphy et al., 2020); and tDCS combined with taVNS, a cranial nerve stimulation, also induced significant improvements on WM in this study. Hence, the effects of other combined stimulation techniques, like tDCS combined with trigeminal nerve stimulation and the underlying mechanism of joint stimulation, are worth investigating in future.

To sum up, combined tDCS and taVNS might be a new non-invasive neuromodulation technique with great clinical prospect, and we would investigate its application in the population with cognitive impairment in future.

## Data Availability Statement

The raw data supporting the conclusions of this article will be made available by the authors, without undue reservation.

## Ethics Statement

The studies involving human participants were reviewed and approved by the Institutional Research Ethics Committee of the Xijing Hospital of the Air Force Medical University. The patients/participants provided their written informed consent to participate in this study.

## Author Contributions

RZ, HD, X-JY, and J-BS were guarantors of integrity of the entire study. RZ, YK, X-JY, and J-BS contributed to study concepts/study design. RZ, Q-QT, Y-PC, Z-YH, M-YC, and F-MW contributed to data acquisition. RZ, Z-YH, CC, Q-QT, Y-PC, and J-BS contributed to data analysis and interpretation. RZ, Z-YH, CC, and J-BS contributed to the manuscript drafting or manuscript revision. All authors contributed to manuscript revision and read and approved the submitted version.

## Funding

This study was funded by the Innovation Team and Talents Cultivation Program of National Administration of Traditional Chinese Medicine (Grant No. ZYYCXTD-C-202004), National Key R&D Program of China (Grant No. 2021YFF0306500), National Science Foundation of China (Grant No. 81901827), Natural Science Basic Research Program of Shaanxi (Grant Nos. 2021JQ-211, 2022JQ-649, and 2021JQ-656), Fundamental Research Funds for the Central Universities (Grant No. XJS201209), and Ph.D. start-up fund of Xi'an Polytechnic University (Grant No. BS201914).

## Conflict of Interest

The authors declare that the research was conducted in the absence of any commercial or financial relationships that could be construed as a potential conflict of interest.

## Publisher's Note

All claims expressed in this article are solely those of the authors and do not necessarily represent those of their affiliated organizations, or those of the publisher, the editors and the reviewers. Any product that may be evaluated in this article, or claim that may be made by its manufacturer, is not guaranteed or endorsed by the publisher.
